# Celebrating a breakthrough: the first-ever FDA-approved treatment for geographic atrophy: a correspondence

**DOI:** 10.1097/JS9.0000000000000477

**Published:** 2023-05-15

**Authors:** Abdul Moiz Khan, Syeda Shahnoor, Hareem Khan, Marium Affendi, Sawsane Ahmad Ghaddar

**Affiliations:** a Department of Internal Medicine, Ayub Medical College, Abbottabad; b Department of Internal Medicine, Dow University of Health Sciences, Karachi, Pakistan; c Faculty of Medical Sciences, Lebanese University, Beirut, Lebanon

*Dear Editor*,Geographical atrophy (GA) is a chronic progressive degeneration of the macula and presents as a late-stage manifestation of age-related macular degeneration^1^. GA affects ~5 million people and is a leading cause of vision loss worldwide. Around one-quarter of cases of legal blindness in the United Kingdom and 20% of cases in North America are attributed to geographical atrophy^2^. GA affecting 90% of all people with age-related macular degeneration (AMD), is characterized by retinal pigment epithelium atrophy with overlying photoreceptor degeneration, hyper transmission into the choroid (increased visibility of the large choroidal vessels) and loss of choriocapillaris^3^. Chronic inflammation caused by factors such as aging, environmental stress, smoking, and genetic predisposition can lead to retinal cell death, contributing to the development of conditions such as AMD and, eventually, GA. The complement cascade is one pathway that leads to this inflammation. The complement system is shown to be a key factor in the pathogenesis of GA^4^. Complement C3 genetic variations are significantly linked to an elevated incidence of both exudative and atrophic AMD. Consequently, complement inhibition is being investigated as a potential therapeutic intervention for GA, and drugs targeting this pathway are currently undergoing clinical trials^4^. Unfortunately, despite these efforts, there have been no approved therapies for this condition until now, making it a major unmet medical need.

The recent development in the field of ophthalmology is the FDA’s (United States Food and Drug Administration) approval, on 17th February 2023, of the first-ever drug, pegcetacoplan (trade name: Empavelli), for the treatment of GA^5^. Primarily used as a drug of choice for paroxysmal nocturnal hemoglobinuria, pegcetacoplan is principally a complement C3 inhibitor that acts by reducing the cleavage and activation of the complement cascade, reducing both extravascular and intravascular hemolysis. The complement system normally is kept in check to avoid damage to cells. Disruption of the complement system seems to be a major contributor to the pathogenesis of GA. Complement activation products have been identified at elevated levels in the plasma of GA patients and locally deposited in ocular tissues. C3 is an attractive target in GA treatment because it is the point of convergence for all three activation pathways. Pegcetacoplan deactivates the pathway and thus prevents the damage done by it^6,7^. The drug was approved after 24-month data obtained from phase 3, multicenter randomized, double masks, sham-controlled trials that compared the efficacy of pegcetacoplan with sham injections. The growth rate of lesions was reduced by 22% with the monthly injection in OAKS, and in DERBY, the monthly injection reduced the growth rate of lesions by 18%, indicating its positive role in slowing the disease progression over time^5^. The drug is given subcutaneously every 30–60 days at a recommended dose of 1080 mg per infusion. It has a half-life of 8 days and reaches steady-state pharmacokinetics after 6–8 weeks.

In conclusion, the FDA’s approval of pegcetacoplan represents a significant milestone in treating GA. This medication’s infrequent dosing represents a substantial convenience for patients. It is imperative that clinicians are made aware of pegcetacoplan and its efficacy, thereby considering it as a viable treatment option for their patients. We recommend updating treatment policies to include pegcetacoplan, and advocate relevant authorities to facilitate payment for the drug, ensuring equitable access to it. We must continue to invest in preventive care measures and research to improve treatment outcomes for this condition.

## Ethical approval and consent to participate

Not applicable.

## Consent for publication

Not applicable.

## Sources of funding

None.

## Author contribution

A.M.K. and S.S.: conceptualization of ideas; H.K., M.A., and S.A.G.: manuscript preparation; H.K., M.A., and S.A.G.: writing of initial drafts; A.M.K. and S.S.: review and editing; A.M.K.: critical review and comments; A.M.K.: data curation. All authors approved the final draft of the manuscript.

## Conflicts of interest disclosure

There are no conflicts of interest.

## Research registration unique identifying number (UIN)

None.

## Guarantor

Sawsane Ahmad Ghaddar, MD, Faculty of Medical Sciences, Lebanese University, Beirut, Lebanon, E-mail: ghaddarsawsane@gmail.com, https://orcid.org/0000-0002-1003-1500

## Data availability statement

No new data were generated.

## Provenance and peer review

Not invited.

## Acknowledgements

None.

## References

[1] EyeWiki. Geographic Atrophy – EyeWiki. Accessed 28 April 2023. https://eyewiki.aao.org/w/index.php?title=Geographic_Atrophy&direction=prev&oldid=75749

[2] Patel PJ Ziemssen F Ng E. Burden of illness in geographic atrophy: a study of vision-related quality of life and health care resource use. Clin Ophthalmol 2020;14:15. 32021065 10.2147/OPTH.S226425 PMC6955611

[3] Fleckenstein M Mitchell P Freund KB. The progression of geographic atrophy secondary to age-related macular degeneration. Ophthalmology 2018;125:369–390. 29110945 10.1016/j.ophtha.2017.08.038

[4] Boyer DS Schmidt-Erfurth U van Lookeren Campagne M. The pathophysiology of geographic atrophy secondary to age-related macular degeneration and the complement pathway as a therapeutic target. Retina 2017;37:819. 27902638 10.1097/IAE.0000000000001392 PMC5424580

[5] AJMC. FDA Approves First Treatment for Geographic Atrophy. Accessed 28 April 2023. https://www.ajmc.com/view/fda-approves-first-treatment-for-geographic-atrophy

[6] DrugBank. Pegcetacoplan: Uses, Interactions, Mechanism of Action | DrugBank Online. Accessed 28 April 2023. https://go.drugbank.com/drugs/DB16694

[7] Liao DS Grossi FV El Mehdi D. Complement C3 inhibitor pegcetacoplan for geographic atrophy secondary to age-related macular degeneration: a randomized phase 2 trial. Ophthalmology 2020;127:186–195. 31474439 10.1016/j.ophtha.2019.07.011

